# SIGNAL: Dataset for Semantic and Inferred Grammar Neurological Analysis of Language

**DOI:** 10.1038/s41597-025-05966-x

**Published:** 2025-10-24

**Authors:** Anna Komissarenko, Ekaterina Voloshina, Anastasia Cheveleva, Ilia Semenkov, Oleg Serikov, Alex Ossadtchi

**Affiliations:** 1 AIRI, Moscow, Russia; 2 Higher School of Economics, Moscow, Russia; 3 Center of Excellence for Generative AI, KAUST, KSA, Riyadh, Saudi Arabia; 4 LIFT, Life Improvement by Future Technologies Institute, Moscow, Russia

**Keywords:** Language, Databases

## Abstract

Recently, the idea of brain-model alignment has been the topic of several influential works. However, most of previous studies were based on datasets collected during regular reading tasks where the subjects were not exposed to processing linguistic incongruencies, and stimuli were not controlled for key linguistic properties. Meanwhile, interpretability studies of Large Language Models pay growing attention to thoroughly designed linguistic tasks based on certain acceptability measures. We present a dataset that contains 600 sentences with a combination of congruent and grammatically or/and semantically incongruent sentences coupled with high density 64-channel EEG recordings of 21 participants. The text stimuli were assessed by native speakers and later used in EEG recording and validation and LLM probing. The validation results proved suitability of the data for future research on brain-model alignment in the linguistic context.

## Background & Summary

Aligning Large Language Models (LLMs) and human brain language processing has been a focus of a growing interest in recent research. Preliminary studies provided evidence that natural language representations captured by LLMs can linearly map onto neuroimaging data representing neural responses during language perception and comprehension^1,2^. However, LLMs performance still falls behind human language abilities supported by complex, dynamic and context-dependent hierarchical multidimensional neural networks^1,3,4^. Hence, research on brain-model alignment represents a rich potential for development of advanced brain-like language-processing systems and further understanding of the principles of language representation implemented in the human brain. However, the majority of previous studies utilized non-standardized linguistic material such as news datasets or narrative stories^5–12^. The choice of linguistic stimuli is crucial when language comprehension is investigated since the lexical-semantic, syntactic, and psycholinguistic properties of words and sentences have been consistently proved to considerably affect neural responses^13–16^. Therefore, a standardized linguistic dataset controlled for the key linguistic properties is required for efficient and consistent future research on brain-model alignment.

Current research on brain-model alignment converges on the concept of predictive coding theory that is shared by both brain and LLMs and results in partial alignment between the two^1,2^. It postulates that the brain is constantly predicting future events and updates information based on reality feedback. Still the predictive mechanisms of the brain within the language processing domain stand beyond LLMs: while LLMs are tuned to make nearby word-level predictions from the given context, the human brain conducts predictions of the hierarchical system of representations over multiple modalities and timescales. Mischler *et al*.^2^ investigated convergence between the hierarchical processing of contextual features in the LLMs and the human brain with functional magnetic-resonance imaging (fMRI) of brain activity. They used sentence embeddings extracted from different layers within the LLMs to predict fMRI brain activations during spoken speech comprehension of the same narratives. The results confirmed that LLM activations can linearly map onto the neural responses. Secondly, enhancement of embeddings with long-range forecast representations of future words improved the prediction of fMRI activations in the language-related brain areas. Finally, the data revealed hierarchical organization of the model-brain predictions: deep-layer representations best modeled middle temporal, parietal, and frontal areas, while the representation emerging in the front-end layers of the LLMs best fitted to the activity in the superior temporal sulcus and gyrus.

It has been hypothesized that the discrepancy between the artificial and biological predictive systems can lie in the ability of the live nervous systems to process information that is not readily predictable from the context at different processing levels^17^. Artificial computational systems like LLMs operate based on the statistical patterns learned from their training data and cannot predict errors in the same way as the brain anticipates and corrects for discrepancies between prediction and reality. Tuckute *et al*.^18^ designed a GPT-based LLM model to examine the extent to which the perceived sentences can modulate brain activity. Firstly, the authors aligned fMRI activations during sentence comprehension with LLM’s sentence representations and showed that neural responses could be successfully predicted from the LLM representations. Next, they used the developed model to generate model-selected sentences, including unnatural poorly predictable ones, that would maximally drive or suppress brain activity. While they found the predictability or the associated surprisal^19^ to be the major predictor of brain activation intensity, they also discovered that the subtle aspects related to the form and meaning of lexical items within a sentence can modulate the neural responses. This highlights the important role of the extended context in which the brain processes the incoming lexical information.

At the same time, Antonello and Huth^3^ argue against the dominant interpretation that predictive coding mechanisms lie in the core of the brain-LLM similarity. The authors suggest that the observed correlations might not reflect a direct correspondence between predictive computational mechanisms but rather stem from the common mapping of the wide range of abstract linguistic features. The core argument is based on the idea that LLMs implicitly learn statistical regularities of linguistic features through their training on massive datasets. These representations can naturally align with the brain’s representations because the brain has also learned these regularities through experience. Therefore, the similarity isn’t necessarily because the brain uses a predictive coding scheme similar to LLMs but rather due to the fact that both systems base their processing on the similar features of the linguistic input.

Recent study by Dentella *et al*.^4^ highlighted that despite the ability of LLMs to produce fluent, semantically coherent outputs, models still lack a real human-like understanding of language. Particularly, LLMs are struggling to map morphosyntactic and pragmatic properties directly onto semantic information during natural language comprehension. They tested their assumptions via systematical assessment of 7 state-of-the-art language models on a series of comprehension questions and compared the results to the humans answers on the same prompts. The results revealed that LLMs were outperformed by humans, indeed with qualitative answers exhibiting clearly non-human error patterns in language comprehension.

Hence, further studies on brain-model alignment could reveal the similarities and discrepancies of the LLM and human language processing mechanisms. These are best exposed using linguistic material with both congruent and incongruent content present at different levels, grammatical and semantic. The following valuable questions could then be asked:

- Whether or not the linguistic properties, such as morpho-syntactic and lexical-semantic features, contribute to the predictive coding mechanisms, and particularly generating the surprisal signal? Which properties are more important?
- Is the contribution of morpho-syntactic parameters to the brain prediction mechanisms similar to that of LLMs?
- What kind of information predicts the alignment of the predictive coding mechanisms in LLMs and human brains?

The corpus of regular and semantically/grammatically incongruent sentences with neurophysiological recordings during the corresponding reading task will be instrumental in answering the above pivotal questions. The incongruence present in the stimuli will facilitate tracking the processing pathway in humans as the mismatch between the expectation and brain response to the sensory information. Exploring the layer- and elementwise representations emerging in the LLM and matching them against those measured in humans is likely to highlight similarities and pinpoint the differences in how the linguistic information is handled in both systems. The obtained findings could not only be used for improving the LLMs^20–24^ but also will contribute to our understanding of neuronal mechanisms supporting the language function. For example, Hollenstein *et al*.^20^ provided a survey of different NLP applications that involve using brain signals. Hale *et al*.^21^ compared neural models with brain signals to choose a better model. Toneva and Wehbe^24^ first probed BERT with brain activity representations and then fine-tuned a model with brain data. The more aligned model showed better results than the original model. Similarly, Hollenstein *et al*.^20^ showed that combining text data and human signals lead to an improvement on downstream tasks. Ren and Xiong^22^ presented a CogAlign framework that suggests a new methodology to combine cognitive and text data for better model representations. Murphy *et al*.^23^ used EEG data to decode part-of-speech tags.

Although there exists a large volume of neuroimaging studies aligning language processing in humans and LLMs, the community is still lacking openly available datasets with carefully created data balanced with respect to the key linguistic properties and high-density brain activity recordings (see Table 1). To our best knowledge, the majority of previous related datasets were based on regular sentences, and there are no datasets exploring the processing of anomalous language data. Precisely, one of the first datasets was collected by Frank *et al*.^6^ who aimed to investigate how language models’ information measures correlates with event related potential (ERP)^25^ amplitude extracted from EEG data. The dataset was based on the UCL corpus of reading times^7^ and consisted of grammatically appropriate sentences extracted from written English narratives. Further, Hollenstein *et al*.^8,9^ collected ZuCo corpus with eye-tracking and EEG recordings of participants reading natural English sentences. The experimental paradigm comprised normal reading tasks and task-specific reading tasks formulated as a linguistic annotation task to various types of information processing such as entity and relation extraction and sentiment analysis with further application to brain-model alignment. Next, Alice Corpus^10^ presented fMRI and EEG recordings of participants listening to the first chapter of Alice in Wonderland in English language. Michaelov *et al*.^5^ collected EEG data of participants reading English sentences with four types of semantic (in)congruence: Best (congruent sentence), Related (low-close completions semantically related to the best completion), Unrelated (low-close completions semantically unrelated related to the best completion), and Implausible completion. The stimuli material was aimed to elicit an N400 component related to the surprisal; the contextual cosine similarity of the target word was measured using LLMs. As for other languages, Frank and Aumeistere^11^ presented a dataset of narrative Dutch sentences and Oseki and Asahara^12^ created a corpus of Japanese newspaper articles both with associated EEG reading data. Still, there is a lack of corpora based on irregular language material aligned w.r.t. morphosyntactic properties coupled with high fidelity neuroimaging recordings available for further research.

**Table 1:** The comparison of our dataset SIGNAL to the existing EEG datasets.

Corpus	N	Stimuli	Validation	EEG parameters
^6^	24	205 English sentences Reg., SS + (partly), WF+, WL-110 comprehension questions	—	32-channels EEG 500 Hz sampling rate No source localization
^8^	12	**1107** English sentences Reg., SS-, WF-, WL + 68 comprehension questions	—	**128**-channels EEG 500 Hz sampling rate No source localization
^9^	18	739 English sentences Reg., SS-, WF-, WL + 42 comprehension questions	—	**128**-channels EEG 500 Hz sampling rate No source localization
^10^	52	84 English sentences Reg., SS+, WF-, WL + 12 comprehension questions	—	61-channels EEG 500 Hz sampling rate No source localization
^11^	34	200 Dutch sentences Reg., SS + (partly), WF+, WL-100 comprehension questions	—	28-channels EEG 500 Hz sampling rate No source localization
^12^	40	20 Japanese newspaper articles Reg., SS+, WF-, WL-No comprehension	—	32-channels EEG **1000 **Hz sampling rate No source localization
^5^	50	500 English sentences Reg. and semantically incongruent, SS-, WF-, WL-110 comprehension questions	+(N = 30)	29-channels EEG 250 Hz sampling rate No source localization
SIGNAL	21	600 Russian sentences **Four types of congruence** **SS**+**, WF**+**, WL + **25 comprehension questions	**+(N = 133)**	64-channel EEG **1000 **Hz sampling rate **Source localization** by calculating inverse operator based on the MNI152 MRI template

*Note*. Reg. refers to regular sentences without incongruent anomalies. SS + /SS− refers to controlled/not controlled syntactic structure. WF + /WF− refers to controlled/not controlled word frequency in the sentences. WL + /WL− refers to controlled/not controlled word length in the sentences. If the corpus was validated, N refers to the number of participants in the validation study.

In the current study, we provide linguistic material of processing of both semantic and grammatical incongruencies. Secondly, the majority of previous studies did not control stimuli for the key lexical-semantic and morphosyntactic properties such as syntactic sentence structure, word frequency, and length. Meanwhile, the quality of stimuli is crucial for neurophysiological experiments: sentence structure, word length, frequency, and position within the sentence significantly influence brain responses^13–16^ and processing of particular words can be even supported by different neural networks depending on the part of speech, argument structure^16,26^. In the current study we provide a dataset with well-controlled stimuli balanced on the key lexical-semantic properties and controlled syntactic structure, which can enable clearer insights into the differences observed in response to various morpho-syntactic properties, enhancing the reliability and interpretability of both LLMs probing and neurophysiological EEG results. Finally, preliminary studies were mostly conducted based on well-studied linguistic material such as English and several European languages. On the other hand, fusional languages, such as Russian, are characterized by a wide range of morphological categories (such as tense, gender, number, person, case) and a complicated inflectional system as opposed to analytical languages that use specific grammatical words rather than inflection (for example, English). The regularities discovered based on such language can shed light on previously unexplored phenomena that may extend to many other fusional languages like Slavic, Romanian, German etc.

We have carefully created a dataset of linguistically plausible sentences and their semantically, grammatically, and semantically-grammatically incongruent counterparts. Our dataset was sourced on plausible congruent Russian sentences extracted from RuSentEval probing suite^27^. We simplified each sentence to one of three structures if possible: Subject + Verb + Object (SVO), Subject + Verb + Adjective + Object (SVAO), or Subject + Verb + Object + Genitive (SVOG). For each congruent sentence, we generated three incongruent counterparts using language model ruBERT: semantically incongruent, grammatically incongruent, and semantically-grammatically incongruent. For each sentence, we estimated words’ frequency and length in phonemes and syllables. We validated the resulting sentences in the online behavioural experiment to prove that (in)congruence type of the sentences is correctly identified by Russian native speakers. Based on the online validation results and assuming words’ key lexical-semantic properties, we obtained the corpus of 600 sentences (150 groups of congruent sentences with their incongruent counterparts) with best annotators’ agreement on the type of (in)congruence.

Further, we conducted two consequent experiments to validate the dataset via neuroimaging evaluation and LLM probing. In the neuroimaging experiment, we collected high-density EEG recordings of humans reading the sentences. We estimated pairwise differences in the EEG evoked responses between (in)congruence types of sentences via statistical tests. Our analysis demonstrates the presence of significant topically organized differences in the EEG data between (in)congruence conditions, thus proving the validity of stimuli types on the neurophysiological level. Secondly, we probed an LLM with the developed corpus of sentences. We performed experiments with LLM to estimate layer-wise contrasts between (in)congruence conditions. Based on our observations, the tested LLM clearly detects the presence of incongruence combined with the observed structure assuming the data validity for further investigations.

Hence, our validation confirmed that the presented dataset is primarily valuable by its quality and well-balanced stimuli parameters as a base for subsequent explorations of the brain-model alignment phenomenon. Firstly, the current dataset is unique as it consists of the well-balanced both regular and anomalous sentences with grammatical and semantic (in)congruence and is controlled for key lexical-semantic parameters. The stimulus includes well-structured and verified language material consisting of groups distinguished by three syntactic structure types and four (in)congruence conditions. In sum, the dataset can be easily divided into four main categories with three subcategories each resulting in a total of 12 well-balanced subcategories. This prospectively enables the RSA^28,29^ (Representational Similarity Analysis) – a powerful yet computationally light tool permitting to align cortical processes with representations emerging in the language models. Secondly, the presented dataset is valuable for the high quality of EEG recordings as measurements of brain activity. This allows for a sufficiently spatially-detailed and time-resolved modelling of neuronal sources to track the dynamics of the prediction errors and to match those against the processes unfolding in a language model. This will help to resolve the reasons behind language models being effective at predicting brain responses to natural language, design novel architectures and training policies to better align artificial agents with human beings and reveal which linguistic aspects dominate in the observed brain-LLM similarities.

In what follows we will present the first dataset comprising carefully selected and crowd-verified sentences with four types of (in)congruence along with the 64-channel EEG recordings from humans reading these sentences in a carefully designed experimental paradigm. The validation results confirmed that the (in)congruence types of stimuli are clearly distinguishable both on the neurophysiological level in humans and in the LLM representations. Thus, the current dataset and the accompanying tools can be beneficial for the potential research into the neural bases of language processing in humans in alignment with NLP performed in LLMs.

## Methods

### Stimuli generation

Our data is based on RuSentEval dataset^27^, the probing suite for Russian language. Firstly, we extracted 2610 sentences from the corpus and marked up each sentence structure using *Natasha* Python library. Further, we reduced each sentence to one of three structures if possible: Subject + Verb + Object (SVO), Subject + Verb + Adjective + Object (SVAO), or Subject + Verb + Object + Genitive (SVOG). In supplementary experiments (Section 2.4.1) we showed that dataset reduction did not affect the observed probing results on the dataset, thus, preserving the general applicability of the data for probing. For each word in the sentences, we extracted frequency from a Russian National Corpus (https://ruscorpora.ru/en/), as well as estimated words’ length in phonemes and syllables.

To obtain sentences of four (in)congruence types, we generated three variants of each sentence with semantical, grammatical, and semantical-grammatical errors by replacing the Object in each sentence. Firstly, we generated semantically incongruent sentences by replacing a target word in a congruent sentence (Object within the sentence structure) with another word. We used the *word2vec* model from *RusVectores* package to generate negative examples and to obtain the furthest word from the original target in a vector space (see Table 2). Then we used congruent and semantically incongruent sentences to generate grammatically and semantically-grammatically incongruent counterparts. To do so, we intentionally put the Object of each sentence in the wrong case or number using the *pymorphy2* (https://pymorphy2.readthedocs.io/en/stable/index.html) Python library (see Table 3).

**Table 2:** Generation of semantically incorrect sentences.

**Table 3:** Generation of grammatically incorrect sentences.

In the result, we obtained sets of sentences that differ from each other only by (in)congruence type of Object argument (precisely, presence or absence of a grammatical/semantic error). For example:

- Congruent sentence
  - *Storony podpisali soglashenie*
  - *‘The parties signed an agreement (accusative)’*
- Semantically incongruent sentence
  - *Storony podpisali detstvo*
  - *‘The parties signed childhood (accusative)’*
- Grammatically incongruent sentence
  - *Storony podpisali soglashenii*
  - *‘The parties signed an agreement (locative)’*
- Semantically-grammatically incongruent sentence
  - *Storony podpisali detstve*
  - *‘The parties signed childhood (locative)’*

In total, we obtained 10440 sentences stimuli of four types of conditions for further evaluation. Based on the results of online validation (see Section 2.2) we obtained 600 sentences (150 congruent ones with three incongruent variants). Congruent sentences were divided into three syntactic structures (50 “congruent” sentences in each group) with sentence arguments balanced in terms of frequency and length between groups (see Table 4). Thus, the corpus contained four main (in)congruence categories with three (syntactic structure) subcategories each resulting in a total of 12 well-balanced subcategories.

**Table 4:** Key parameters of sentences between groups.

Sentence structure	Sentence argument	Length (syll.), M	Length (syll.), SD	Frequency (IPM), M	Frequency (IPM), SD
Subject - Verb - Object	Subject	3.18	0.98	102.75	135.1
Subject - Verb - Object	Verb	3.64	0.94	113.02	158.14
Subject - Verb - Object	Object	3.3	1.19	120.05	167.27
Subject - Verb - Adjective - Object	Subject	3.2	1.0	162.41	424.47
Subject - Verb - Adjective - Object	Verb	3.5	0.97	154.37	179.53
Subject - Verb - Adjective - Object	Object	3.26	1.39	94.79	86.98
Subject - Verb - Adjective - Object	Adjective	4.0	1.13	141.28	274.98
Subject - Verb - Object - Genitive	Subject	3.6	1.27	117.35	185.7
Subject - Verb - Object - Genitive	Verb	3.76	0.89	123.96	200.2
Subject - Verb - Object - Genitive	Object	3.32	1.31	129.81	207.81
Subject - Verb - Object - Genitive	Genitive	3.78	1.38	137.51	248.08

Note. Sentence structure - syntactic structure of stimuli sentences. Sentence argument - syntactic argument of the sentences. Length - length of the argument lemma in syllables. Frequency - frequency of the argument lemma based on the Russian National Corpus (https://ruscorpora.ru/en/).

### Online validation experiment

To collect humans’ acceptability judgements for the generated sentences, we used a crowdsourcing platform Toloka (https://toloka.ai/). The workers were asked to assess whether a given sentence is congruent, or it contains either a semantic, grammatical error, or both types of error (see the example of web interface and instructions at Supplementary Information). The assessment was only available for people who listed Russian as their native language and come from Russia, Belarus, Ukraine or Kazakhstan (based on their phone number).

Before the assessment, participants were presented an annotation instruction and trained on sample sentences. During the assessment, the quality was controlled via control sentences. Both training and control sentences were preliminary annotated by the authors. For additional quality control, we monitored the consistency of participants’ responses compared to others. Highly inconsistent responses led to temporal participant exclusion, assuming insufficient training on the sample sentences. To ensure a diverse pool of participants, we limited the amount of money one participant can earn.

Thus, we use the following constraints for workers to ensure faithful annotation:

- Participants were only allowed to complete an assessment if they annotated the 26 sample sentences with at least 90% accuracy during the training;
- Participants were suspended from the assessment for 2 days if they earned 5 dollars or more (as they annotated too many sentences);
- Participants were banned for 2 hours if they skipped 7 sentences or more or the quality on control sentences was less than 50% out of 4 recent responses;
- Participants were banned for 3 days if 30% of their responses were different from other annotators.

In total, 133 participants annotated sentences with an average of 6.95 sentences per person and at least 3 independent crowd workers per each sentence. Participants were paid $0.014 for each sentence. Given economic constraints and initial goal to get 150 sentences per condition, we did not annotate the whole machine-generated set. The overall number of annotated sentences was 1465 that were later filtered out if less than 75% of crowd workers did not correctly annotate the sentence. After filtration, we obtained 600 sentences consisting of 150 groups of sentences (congruent sentences and its counterparts with semantic, grammatical, and semantic-grammatical errors).

### EEG experiment

#### Participants

Data of 21 participants (*M*_*age*_ = 21.76, *SD* = 3.66, female = 16) were collected in the experiment. All participants were right-handed native Russian speakers, did not have a linguistic background and were not proficient speakers of any foreign language other than English. They had normal or corrected vision and hearing, no history of neurological deficits or language-related impairments. All participants were paid a standard rate of $2.7 per hour. Participants signed a written informed consent form prior to taking part in the research and completed the screening questionnaire form to estimate language proficiency and reading skills and to reveal any contraindications. To ensure participant privacy and data integrity, the collected EEG and behavioral data were anonymized through the generation of a unique numerical identifier. This identifier was then systematically applied to name all files pertaining to the participant. Participants’ names and any other identifying data were not recorded on any medium, apart from their signed informed consent forms for study participation. The study was approved by the HSE Committee on Interuniversity Surveys and Ethical Assessment of Empirical Research under protocol number 80 on February 7, 2022.

According to the screening questionnaire, all participants had high proficiency in Russian, as they had good or excellent grades in school in Russian language and all participants were well-read. All participants had at least 11 years of education, which is equal to upper secondary education (*M*_*age*_ = 14.26 years of education, *SD* = 1.74).

#### Procedure

The experiment started with a participants’ preparation and montage of electrodes. Participants were fitted with a 64-electrode cap (AntiCap, BrainProducts, German) in a sound attenuating, electrically shielded booth with a monitor and a keyboard. Electrodes were positioned on the scalp according to the International 10–20 system with a Cz electrode used as a reference. EEG data were collected using an ActiCap electrode system (BrainProducts, Germany) and actiCHamp amplifier (BrainProducts, Germany) at 1000 Hz sampling rate with impedance below 10 kΩ.

**Fig. 1:**
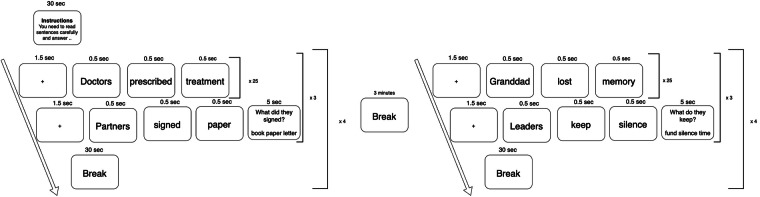
The outline of the experiment.

The experimental task consisted of 600 sentences described in Section 2.1. Additionally, 25 control sentences with context-related questions were included into the task to control for participants’ attention. Participants were instructed to read sentences attentively from the screen and to answer sentence-related questions after a part of the sentences. The task was presented on the screen in the beginning of the experiment for a second time. During the experiment, each sentence started with a fixation cross lasting for 1.5 seconds. Then sentences appeared on the screen word by word with a speed one word per 0.5 seconds close to natural reading speed^30^. The order of (in)congruence type and positions of filler sentences were randomized in order to prevent participants from getting a clue of the experiment. Counterparts of similar sentences (differing only by (in)congruence type) were set on a maximum distance so there were at least 100 other sentences between two similar items. There were six 30-second breaks during the experiment after every 75 sentences (every two-three minutes) and a long three-minute break in the middle of the experiment to check the impedance of the electrodes and give participants time to rest and refresh their attention. The total duration of the experiment was two hours including preparation time. The outline of the experiment is visualized in Fig. 1.

#### EEG preprocessing and analysis

Data processing was performed via *MNE* Python library^31^. A low-pass zero-phase filter with a 30-Hz cutoff, and a high-pass filter with a 0.1-Hz were applied to the EEG recordings. Based on the spectral characteristics, noisy/flat channels were interpolated. Independent component analysis (ICA) was performed to remove ocular artifacts such as horizontal and vertical eye movements. The data were divided into epochs containing only the target stimuli recordings, including 400 ms pre-stimulus and 800 ms post-stimulus interval. The raw EEG data included mark-up with a unique identifier of each sentence and a (in)congruence condition of the target word. Baseline correction was applied to the epochs using 0-th order detrend computed over the pre-stimulus (−400 ms - 0 ms) interval.

Statistical analysis was aimed to reveal statistically different temporal-spatial clusters of EEG data between four stimuli (in)congruence conditions. To do so, we firstly obtained ERP data by averaging EEG response between participants and conditions. Secondly, we computed mean values and standard deviations of ERP to do z-score estimation to assess the presence of differences between each pair of conditions. Grand-average data were obtained via averaging z-scores between participants. To estimate a statistical significance of results, a simple permutation test was performed on the ERP maps, p-values were combined with a Fisher’s combined probability test. Benjamini-Hochberg^32^ procedure was applied to correct for multiple comparisons using the false discovery rate (FDR) correction procedure with the critical alpha level set to α = 0.023.

In order to ensure that EEG responses in different (in)congruence conditions within one sentence group were not affected by priming effect, we compared ERP data between consecutive occurrences within similar sentence groups (e.g., first and second occurrences of similar sentences). To do so, we applied singular value decomposition (SVD) to the averaged ERP within the whole dataset. Then we extracted ERP of the first, second, third, and fourth occurrences within each sentence group for each participant so that the resulting data included sentences of different (in)congruence types.. We averaged ERP between consecutive occurrences and projected them onto the subspace defined by the first four singular vectors (corresponding to the largest singular values) obtained from the SVD. Finally, we computed correlation matrices between resulting projections related to each singular vector.

Finally, we performed inverse modelling using MNE python library to estimate the cortical sources underlying the pairwise (in)congruence condition differences observed in sensor space. The activity of the sources was reconstructed using an inverse kernel obtained with a dynamic statistical parametric maps (dSPM) method. The noise covariance was computed from the baseline, the signal-to-noise ratio (SNR) parameter was set to 3. We contrasted the resulting source estimates to obtain pairwise difference between (in)congruence conditions. The latencies were set according to ERP peaks in the components’ window.

### LLM probing experiment

The LLM probing validation was aimed to verify whether the LLM exhibits differential activation in response to different types of errors in the dataset to confirm the dataset’s suitability for aligning LLM representations with EEG data. As EEG recordings were made on a carefully curated subset of the larger dataset derived from RuSentEval, we conducted LLM probing on both the initial dataset and the curated subset.

In the experiment, we collected activations of each layer of the ruBERT LLM^33^, de-facto standard LLM adapted for the Russian language. The model follows the standard BERT architecture of 12 layers, each consisting of 768 neurons. Probing experiments were performed using the Google Colaboratory free compute service and took approximately one hour of computation in total. Following Conneau and Kiela^34^, we applied logistic regression to a binary pairwise classification of sentences (in)congruence types based on LLM layer-wise sentence representations. As sentence representations, we used contextualized vectors of the word bearing the congruence/incongruence of the relative sentence. If a word consisted of several sub-tokens, the respective embeddings were averaged (see more details on tokenization safety-check below). We mitigated the effect of random seeds in our small-scale experiments by performing each experiment 20 times.

#### Tokenization study

When creating incongruent sentences, the number of tokens was not changed in most cases compared to the original sentence according to the tokenizer used in the model. Only in 7.5% of all cases, semantically incongruent sentences were one token longer than the original ones. Similarly, only in 8.5% of all cases, grammatically incongruent sentences were one token longer than the original ones. Thus, the sentence length in tokens was unlikely to be a determining factor for a probing model to make a decision about (in)congruence type of the sentence.

## Data Record

The dataset is available at Hugging Face^35^ and associated code at https://github.com/AIRI-Institute/SIGNAL. The “stimuli.csv” file contains a stimuli dataset with the respective linguistic properties. The columns are described in Table 5.

**Table 5:** Structure of the stimuli dataset.

Column name	Dtype	Description
sentence_id	int	Unique identifier of congruent sentence
sentence	str	Sentence stimuli including congruent and incongruent variants
congruent	str	Original congruent sentence for each respective congruent and incongruent counterparts
structure	str	Syntactic structure of the sentence
length	int	Sentence length in words
target	str	Type of (in)congruence condition (“normal” - congruent, “semantics” - semantically incongruent, “grammar” - grammatically incongruent, “semantics_grammar” - semantically-grammatically incongruent)
position	int	Position of the word with semantic and/or grammatical error within incongruent sentences
most_popular	str	Most popular answer ((in)congruence condition) within assessors in the online validation experiment
percent	float	Percent of accessors who gave the answer specified in the “most_popular” column in the online validation experiment
semantics_grammar	float	Percent of accessors who evaluated the sentence to be semantically-grammatically incongruent in the online validation experiment
semantics	float	Percent of accessors who evaluated the sentence to be semantically incongruent in the online validation experiment
grammar	float	Percent of accessors who evaluated the sentence to be grammatically incongruent in the online validation experiment
no	float	Percent of accessors who evaluated the sentence to be congruent in the online validation experiment
unknown	float	The percentage of answers in the online validation experiment where sentences were judged incongruent, with the identified error type being neither semantic nor grammatical.
{subject/verb/object/gen/adj}	str	Sentence subject/verb/object/gen/adj argument
{subject/verb/object/gen/adj}_lemma	str	Lemma (initial form) of sentence subject/verb/object/gen/adj argument
{subject/verb/object/gen/adj}_length	int	Length of sentence subject/verb/object/gen/adj argument in syllables
{subject/object/gen/adj}_gender	str	Linguistic gender of sentence subject/object/gen/adjargument
{subject/verb/object/gen/adj}_ipm	float	Frequency (Instances Per Million) of sentence subject/verb/object/gen/adj argument

Note. Column - column names. Dtype - data type within the columns. Description - description of the information included in the columns.

The EEG data are available in the “.fif” and “.set” formats. The files like “pX_epochs.set” and “pX_epochs.fif” include the epoched EEG data in the “.set” and “.fif” formats respectively; “X” in the filename refers to the participant’s unique identifier.

Each file contains epoched data with the EEG response only to the target (in)congruent word, but not to the whole sentence. Event names refer to the type of (in)congruence and syntactic structure of the related sentence. Table 6 describes (in)congruence types and sentence structure of events.

**Table 6:** Description of the events in the EEG data.

Event name	(In)congruence condition	Sentence structure
Stimulus/S 1_1	Congruent	Subject - Verb - Object
Stimulus/S 1_2		Subject - Verb - Adjective - Object
Stimulus/S 1_3		Subject - Verb - Object - Genitive
Stimulus/S 2_1	Semantically incongruent	Subject - Verb - Object
Stimulus/S 2_2		Subject - Verb - Adjective - Object
Stimulus/S 2_3		Subject - Verb - Object - Genitive
Stimulus/S 3_1	Grammatically incongruent	Subject - Verb - Object
Stimulus/S 3_2		Subject - Verb - Adjective - Object
Stimulus/S 3_3		Subject - Verb - Object - Genitive
Stimulus/S 4_1	Semantically-grammatically	Subject - Verb - Object
Stimulus/S 4_2		Subject - Verb - Adjective - Object
Stimulus/S 4_3		Subject - Verb - Object - Genitive

## Technical Validation

The statistical analysis was aimed to ensure our dataset satisfies the necessary conditions for that kind of data. Firstly, we estimated EEG data to reveal distinguishable differences between stimuli (in)congruence types on neurophysiological level. Secondly, we estimated whether LLMs intermediate activations allow distinguishing these groups of sentences.

### Online experiment validation

Based on online validation results, we selected 600 sentences consisting of 150 groups of sentences (congruent sentences and its counterparts with semantic, grammatical, and semantic-grammatical errors) which error types were correctly identified by more or equal than 75% participants. Consequently, the online validation approved that error types of all sentence stimuli were clearly identified by human participants on the behavioral level.

### EEG experiment validation

**Fig. 2:**
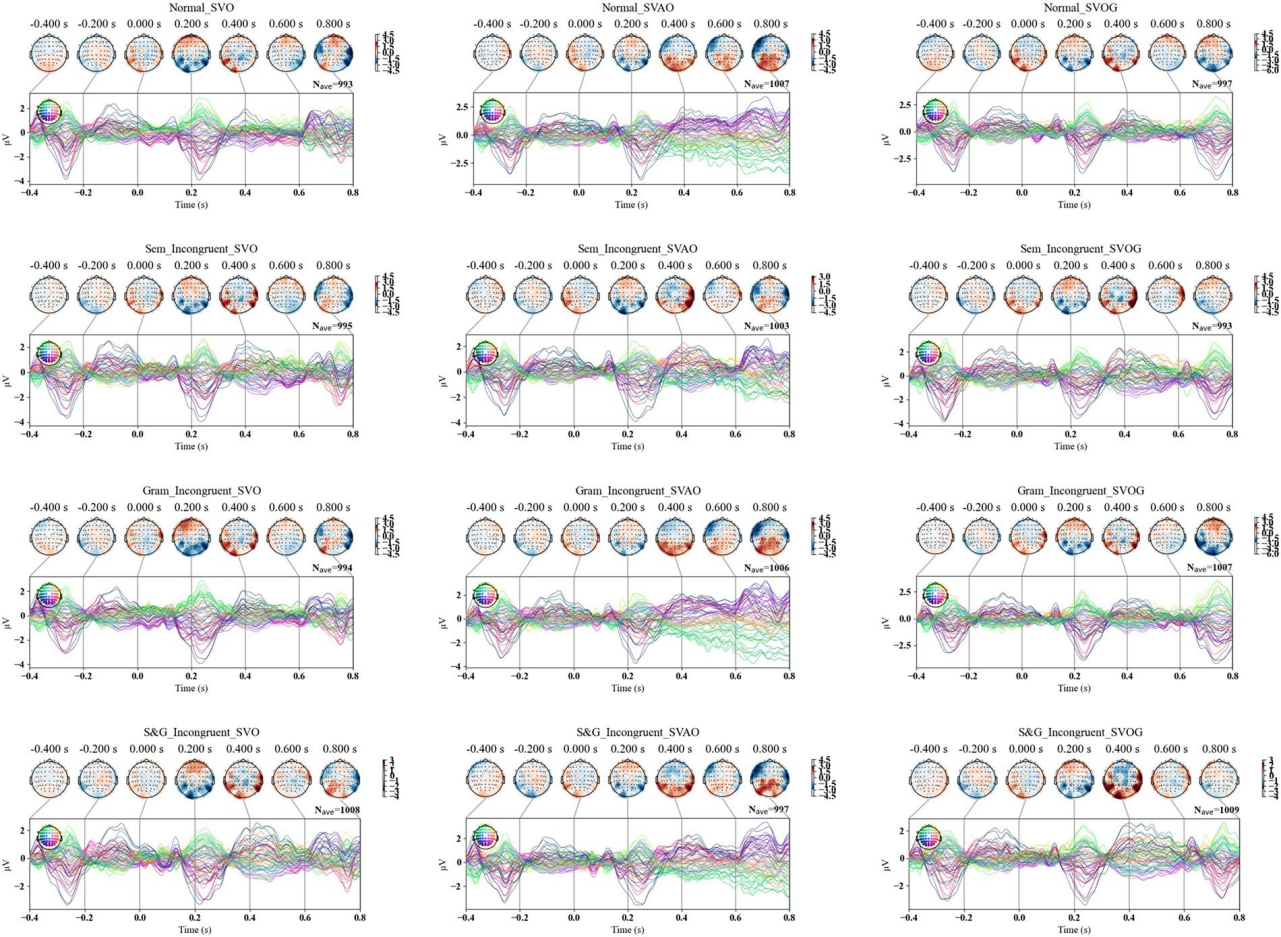
Averaged ERP across participants, sentences, and conditions. Note. The plots represent averaged ERP across participants, (in)congruence conditions, and sentences. The respective time-intervals are presented over a 200 ms time window. “Normal” – congruent condition; “Sem_Incongruent” - semantically incongruent condition; “Gram_Incongruent” - grammatically incongruent condition; “S&G_Incongruent” - semantically and grammatically incongruent condition. “SVO” - “Subject - Verb - Object” sentence structure; “SVOG” - “Subject - Verb - Object - Genitive” sentence structure; “SVAO” – “Subject - Verb - Adjective - Object” sentence structure.

**Fig. 3:**
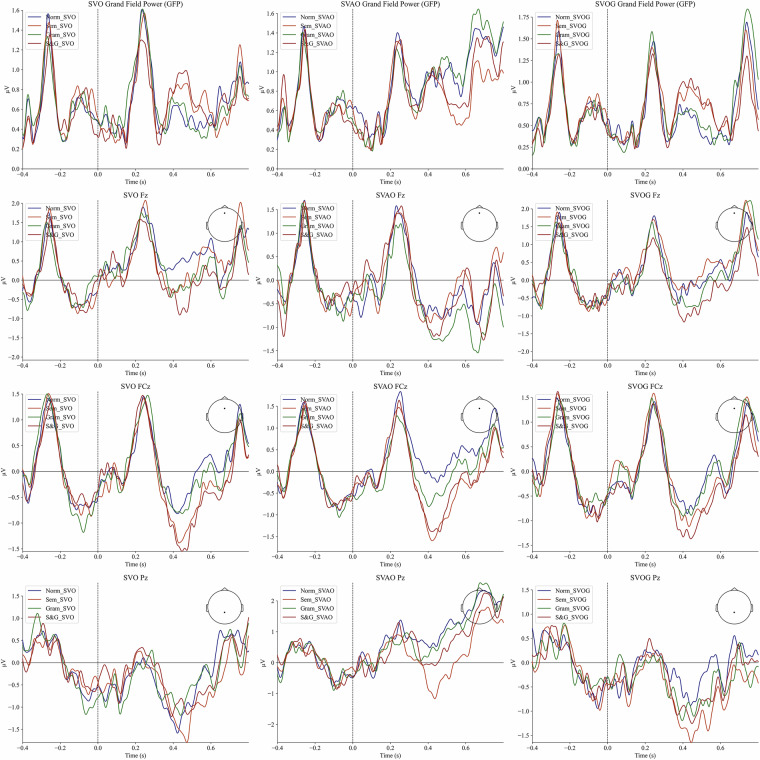
Global Field Power and ERP for central electrodes Fz, FCz, Pz averaged across participants, sentences, and conditions. Note. The plots represent the averaged ERP across (in)congruence conditions, participants and sentences. The respective time-intervals are presented over a 200 ms time window. “Norm_X” – congruent condition with the X sentence structure. “Sem_X” - semantically incongruent condition with the X sentence structure. “Gram_X” - grammatically incongruent condition with the X sentence structure. “S&G_X” - semantically-grammatically incongruent condition with the X sentence structure. “SVO” - “Subject - Verb - Object” sentence structure. “SVAO” - “Subject - Verb - Adjective - Object” sentence structure. “SVOG” - “Subject - Verb - Object - Genitive” sentence structure.

Participants’ performance in a behavioral control task was high (*M* = 0.81, *SD* = 0.20). Figure 2 represents averaged ERP between participants and (in)congruency conditions. Figure 3 represents averaged ERP in central channels Fz, FCz, Pz, as well as Global Field Power.

According to Figs. 2, 3, all (in)congruence conditions elicited negative (pointing up) deflection in the interval around 300 ms post stimulus with a peak at approximately 400 ms post stimulus. This may represent the expected N400 component traditionally associated with processing of semantic violations^36^. The amplitudes of N400 peak appear to be higher in the semantically and semantically-grammatically incongruent conditions compared to the congruent and grammatically incongruent ones.

At the same time, the data revealed a mild positive deflection peaking around 600 ms post stimulus in all conditions. This can be related to P600 effect^36,37^ associated with processing of syntactic violations and unexpected sentence structures. The amplitude of p600 is less pronounced due to the visual processing of the next stimulus (fixation cross in the sentences with “Subject - Verb - Object” and “Subject - Verb - Adjective - Object” and word stimulus in sentences with “Subject - Verb - Object - Genitive” structure) presented after 500 ms. The chosen 500 ms interval between words presentation leads to masking the late response components, however this constraint is imposed by the natural reading natural reading speed requirement^30^ which we think is important. Late ERP components can be recovered using a deconvolution method such as the xDawn^38^ algorithm to enhance evoked potentials.

**Fig. 4:**
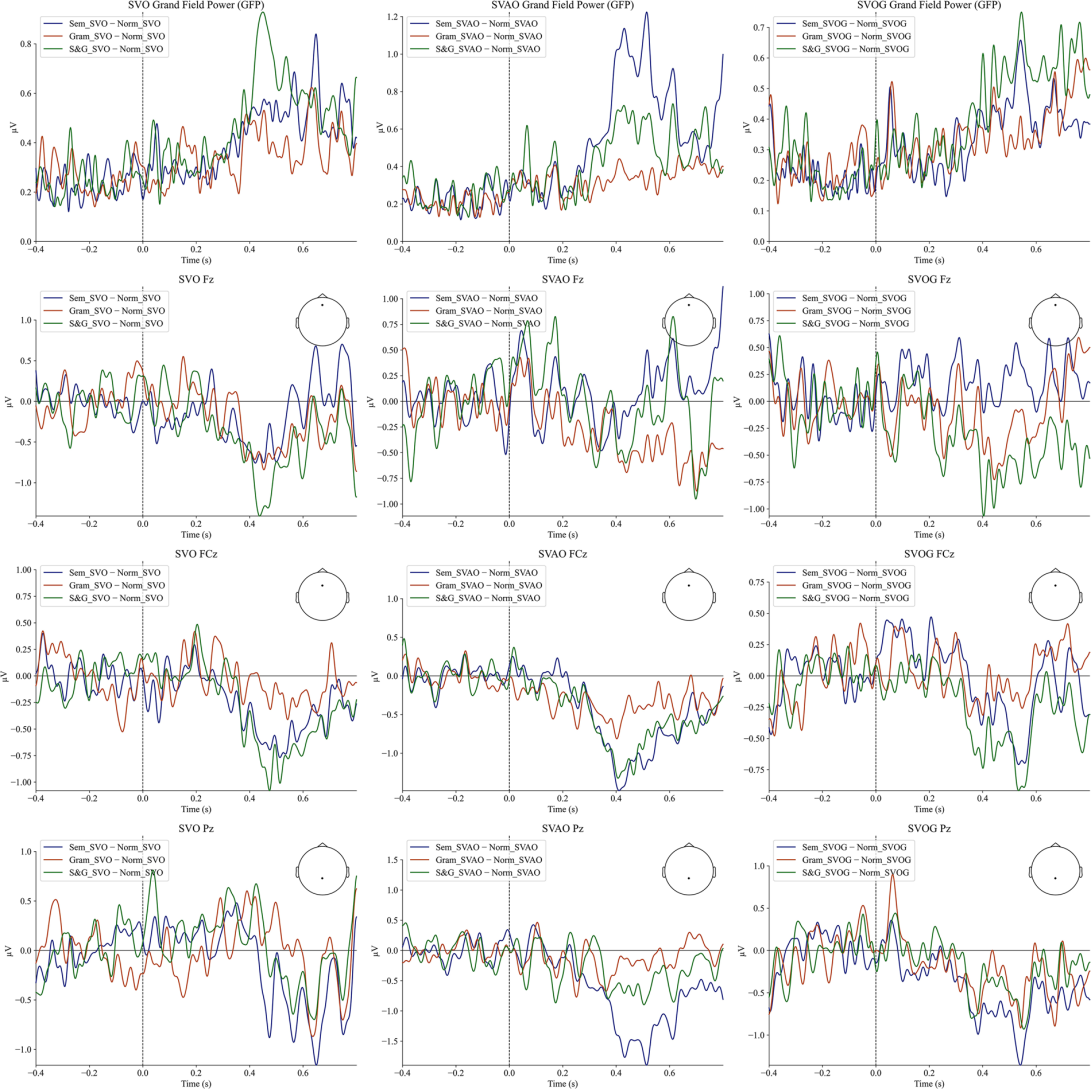
The difference in averaged ERPs: incongruent conditions minus congruent conditions. Note. The plots represent the pairwise difference of averaged ERP between incongruent and congruent conditions, and sentences. The respective time-intervals are presented over a 200 ms time window. “Sem_X - Norm_X” – difference between semantically incongruent and congruent conditions with the X sentence structure. “Sem_X - Norm_X” – difference between semantically incongruent and congruent conditions with the X sentence structure. “Gram_X - Norm_X” – difference between grammatically incongruent and congruent conditions with the X sentence structure. “S&G_X - Norm_X” – difference between semantically-grammatically incongruent and congruent conditions with the X sentence structure. “SVO” - “Subject - Verb - Object” sentence structure. “SVAO” - “Subject - Verb - Adjective - Object” sentence structure. “SVOG” - “Subject - Verb - Object - Genitive” sentence structure.

Figure 4 represents the averaged ERP difference between incongruent and congruent conditions.

**Fig. 5:**
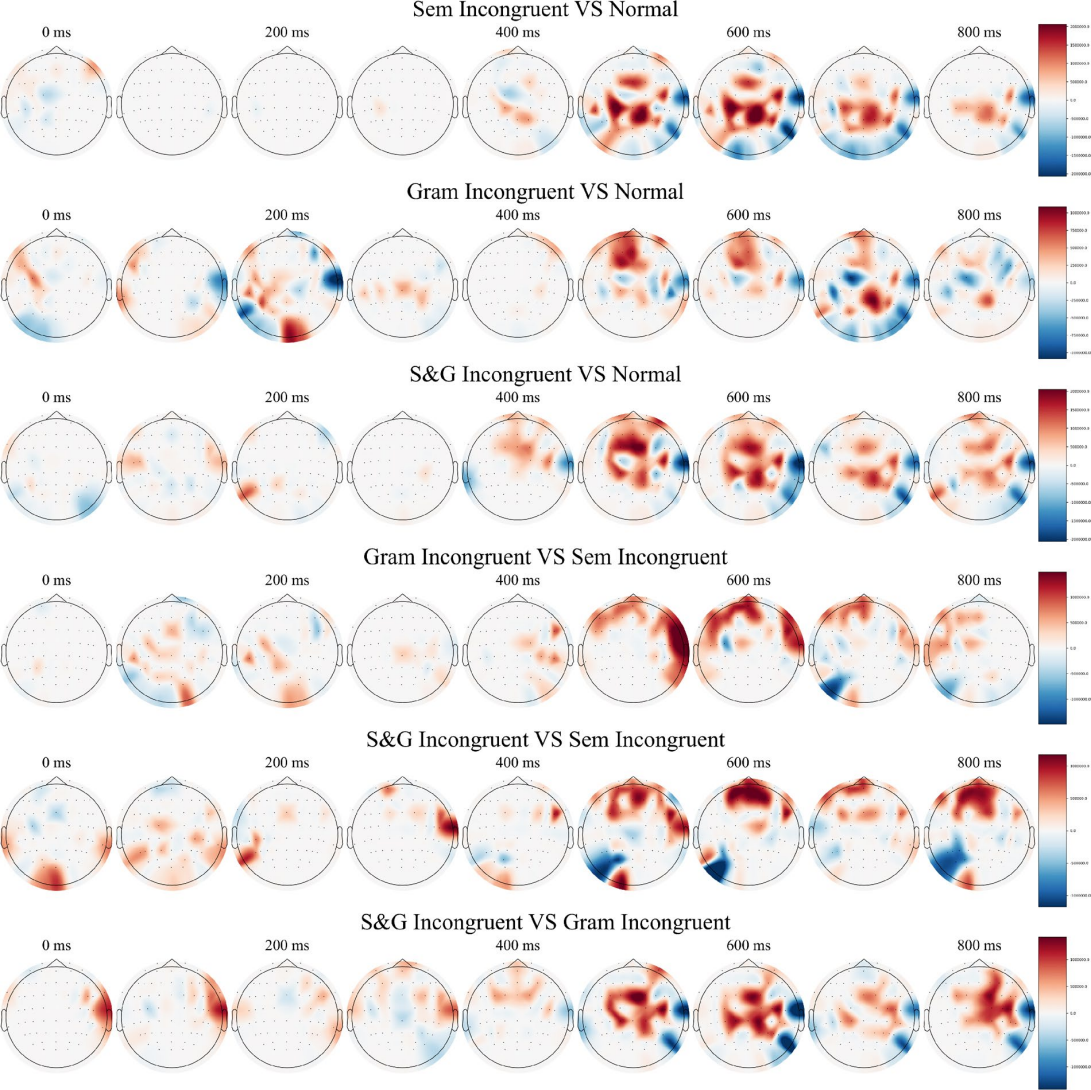
Time resolved topographical plots of z-scores between conditions. Note. The data represents significantly different pairwise z-scores between (in)congruence conditions obtained via z-score estimation and permutation tests corrected for a false-discovery rate with alpha set to α = 0.023. The respective time-intervals are presented over a 100 ms time window. Normal – congruent condition. Sem Incongruent – semantically incongruent condition. Gram Incongruent – grammatically incongruent condition, S&G Incongruent – semantically-grammatically incongruent condition.

Figure 5 represents a topography of statistically significant z-scores based on z-scores estimation and permutation tests corrected for a false-discovery rate.

According to Fig. 4, the greatest amplitude ERP difference in between conditions is observed in the interval of 400–600 ms post-stimulus. Specifically, the amplitude difference for semantic and semantic-grammatical errors is larger than for grammatical errors. Taken together, these findings strongly suggest an association with the N400 effect^39^, a well-established marker for semantic anomaly processing. According to Fig. 5, the results of z-statistics estimation revealed significant differences in all pairs of conditions. The most pronounced divergence between conditions was again revealed in the 400–600 ms interval post-stimulus, concentrated in the central cortical regions and the right frontal and temporal areas contralateral to the established Broca’s and Wernicke’s areas classically associated with language processing^40^. The revealed ERP difference between conditions is likely attributable to the N400 effect which can be asymmetrically distributed over the cortex^39^.

**Fig. 6:**
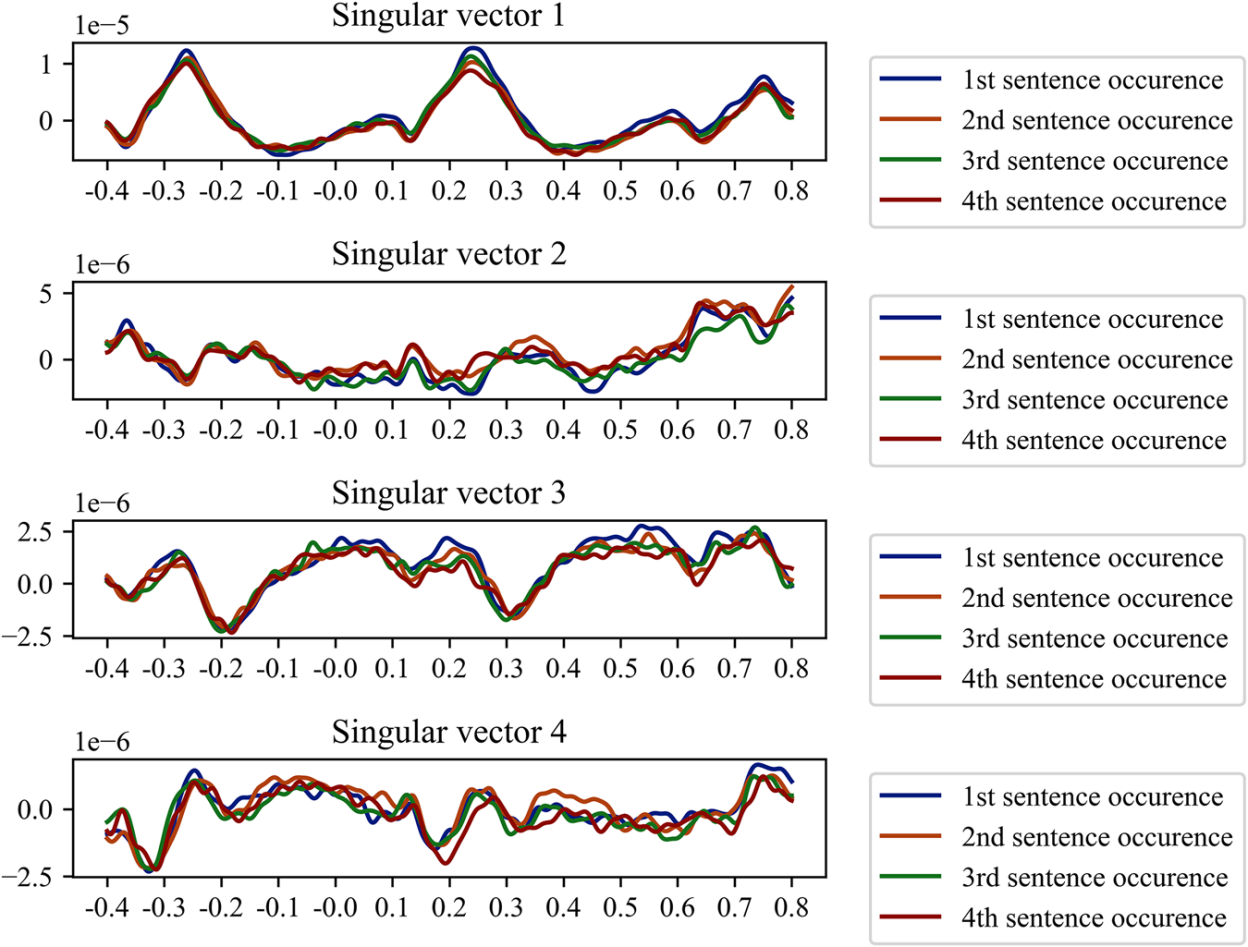
Averaged ERP projections onto first four singular vectors between consecutive occurrences within sentence groups.

**Fig. 7:**
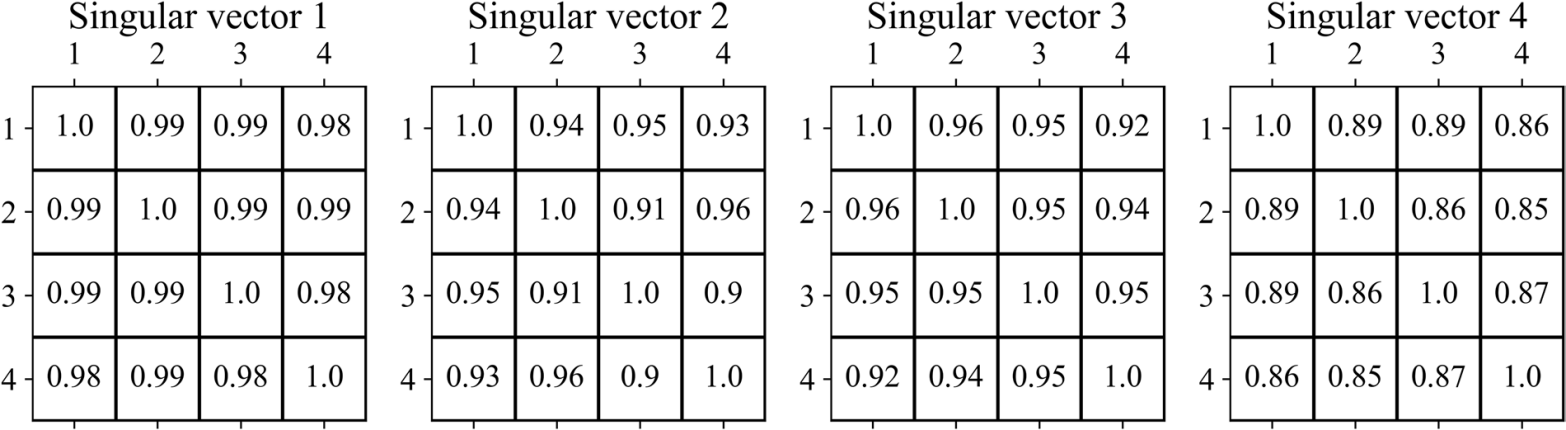
Correlation coefficients of ERP projections onto the first four singular vectors obtained from SVD between consecutive occurrences within sentence groups. Note. The matrices represent pairwise correlation coefficients between ERP projections of consecutive occurrences within similar sentence groups onto the subspace defined by first four singular vectors obtained via SVD. Rows and columns refer to the first, second, third, and fourth occurrences within sentence groups.

Results of priming effect analysis revealed high correlation between ERP amplitude related to consecutive occurrences within similar sentence groups. Figure 6 represents time-resolved projections of the averaged ERP for each consecutive occurrence within sentence groups onto the first four right singular vectors resulting from singular value decomposition of averaged ERP data within the whole dataset. While the response components remain largely intact we do observe a small decreasing trend of the earliest response amplitude in component 1 as participants moved deeper into the experiment. To gauge the overall ERP similarity we computed pairwise correlation coefficients between ERP projections of consecutive sentence occurrences onto each singular vector, see Fig. 7.

According to the results of priming effect analysis, the ERP projections of consequent occurrences within sentence groups onto the first four vectors obtained via SVD decomposition are highly correlated. This may suggest that the EEG response remained unaltered upon consecuitive occurrences within similar sentence groups. Therefore, these sentences were separated temporally for a sufficient distance and filled with other stimuli, minimizing the possible priming effect.

**Fig. 8:**
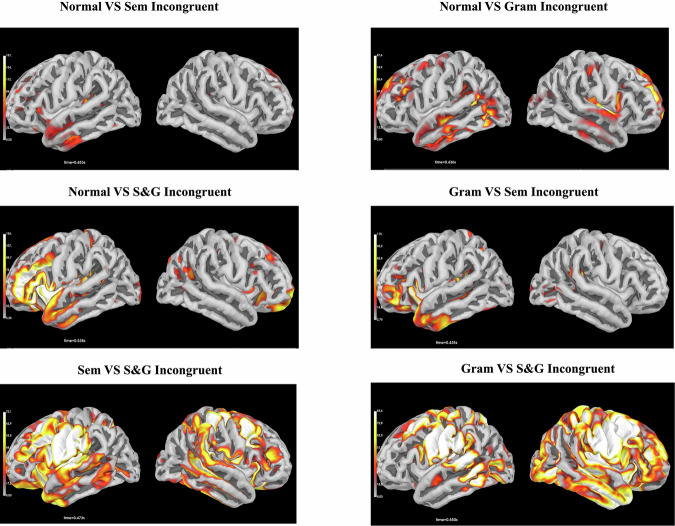
Cortical sources contrasting ERPs between different (in)congruence conditions. Note. Cortical profiles correspond to the peak amplitude latency of the ERP difference between the indicated pair of conditions. ERP contrast between normal and semantically incongruent conditions peaks at 453 ms post stimulus; between normal and grammatically incongruent conditions - at 436 ms post stimulus; between normal and semantically-grammatically incongruent conditions - at 538 ms post stimulus; between grammatically and semantically incongruent conditions - at 435 ms post stimulus; between semantically and semantically-grammatically incongruent conditions - at 473 ms post stimulus; between grammatically and semantically-grammatically incongruent conditions - at 550 ms post stimulus.

Results of source localization are represented at Fig. 8. Cortical sources contrasting processing of congruent and semantically incongruent sentences were observed in the left superior temporal gyrus peaking at 453 ms post-stimulus. The ERP contrasts between congruent and grammatically incongruent sentences were observed in the left superior and posterior temporal areas and in the middle and superior frontal gyri peaking at 436 ms post-stimulus. The contrasts between congruent and semantically-grammatically incongruent areas were observed in both left frontal and temporal cortical areas peaking at 538 ms post-stimulus. The contrasts between semantically and grammatically incongruent areas were observed in both left superior frontal and superior temporal cortical areas peaking at 435 ms post-stimulus. The contrasts between semantically incongruent and semantically-grammatically incongruent areas were observed bilaterally in the frontal and temporal cortical areas peaking at 473 ms post-stimulus. The contrasts between grammatically incongruent and semantically-grammatically incongruent areas were observed bilaterally in the frontal, temporal, and parietal cortical areas peaking at 550 ms post-stimulus.

According to the current evidence, language network, and particularly language comprehension and semantic processing, is supported by extensive hierarchical networks located at frontal and temporal areas in the left hemisphere^16,40^. Thus, the found cortical sources were found in brain areas relevant to language comprehension, thus proving the validity of the stimuli material on the neurophysiological level.

To summarize, EEG data revealed significant differences between semantically, grammatically and semantically-grammatically incongruence conditions, with a pronounced N400 effect peaking around 400 ms post-stimulus, commonly associated with semantic violations processing. This effect was most pronounced in central, right frontal and temporal regions contralateral to Broca’s and Wernicke’s areas. While P600 effects were also observed, their late components were potentially masked by the next stimulus response. Nonetheless, this limitation was necessitated to maintain a natural reading speed to make the experiment more naturalistic and to align them with real-world language processing.

### LLM probing validation

**Fig. 9:**
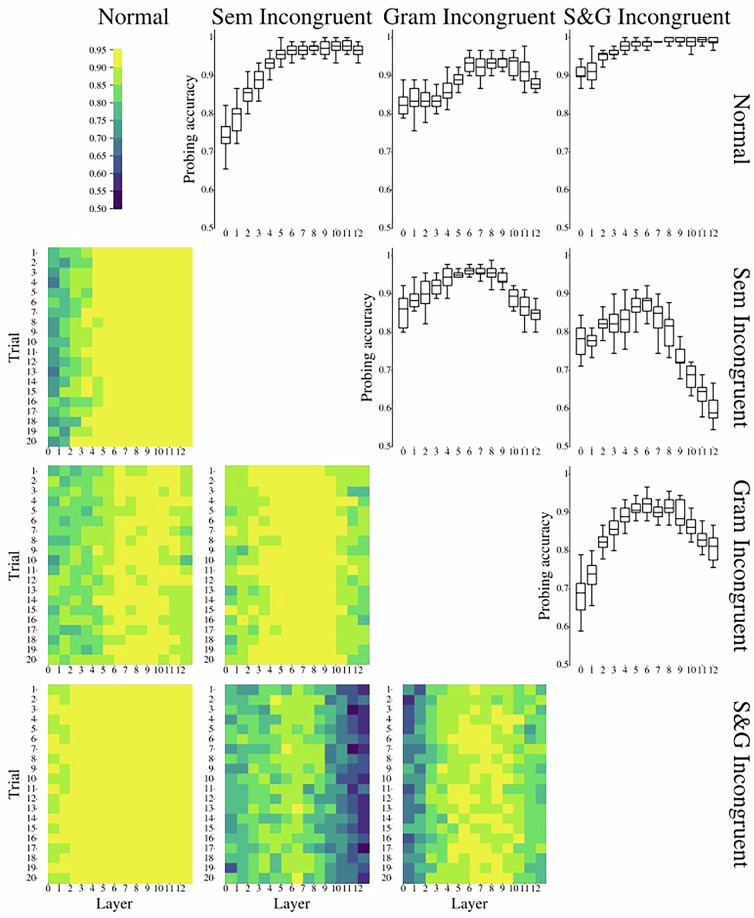
Results of layer-wise probing validation of the current dataset. Note. The lower matrix triangle represents the accuracy scores for the pairwise (in)congruence types classification obtained by applying logistic regression. The upper matrix triangle represents boxplots of the obtained accuracy scores; each boxplot shows the distribution of accuracy scores within 20 independent runs for each LLM layer and each pair of conditions. Normal – congruent condition. Sem Incongruent – semantically incongruent condition. Gram Incongruent – grammatically incongruent condition, S&G Incongruent – semantically-grammatically incongruent condition.

Figure 9 represents the results of LLM probing experiments. We find middle-to-high layers’ activations to be most prominent to detect (in)congruence types in a sentence. Semantically incongruent sentences were easier to detect than grammatically incongruent ones. At the same time, semantically-grammatically (S&G) incongruent sentences were the easiest to distinguish from congruent ones and were the hardest to distinguish from both semantically and grammatically incongruent sentences.

**Fig. 10:**
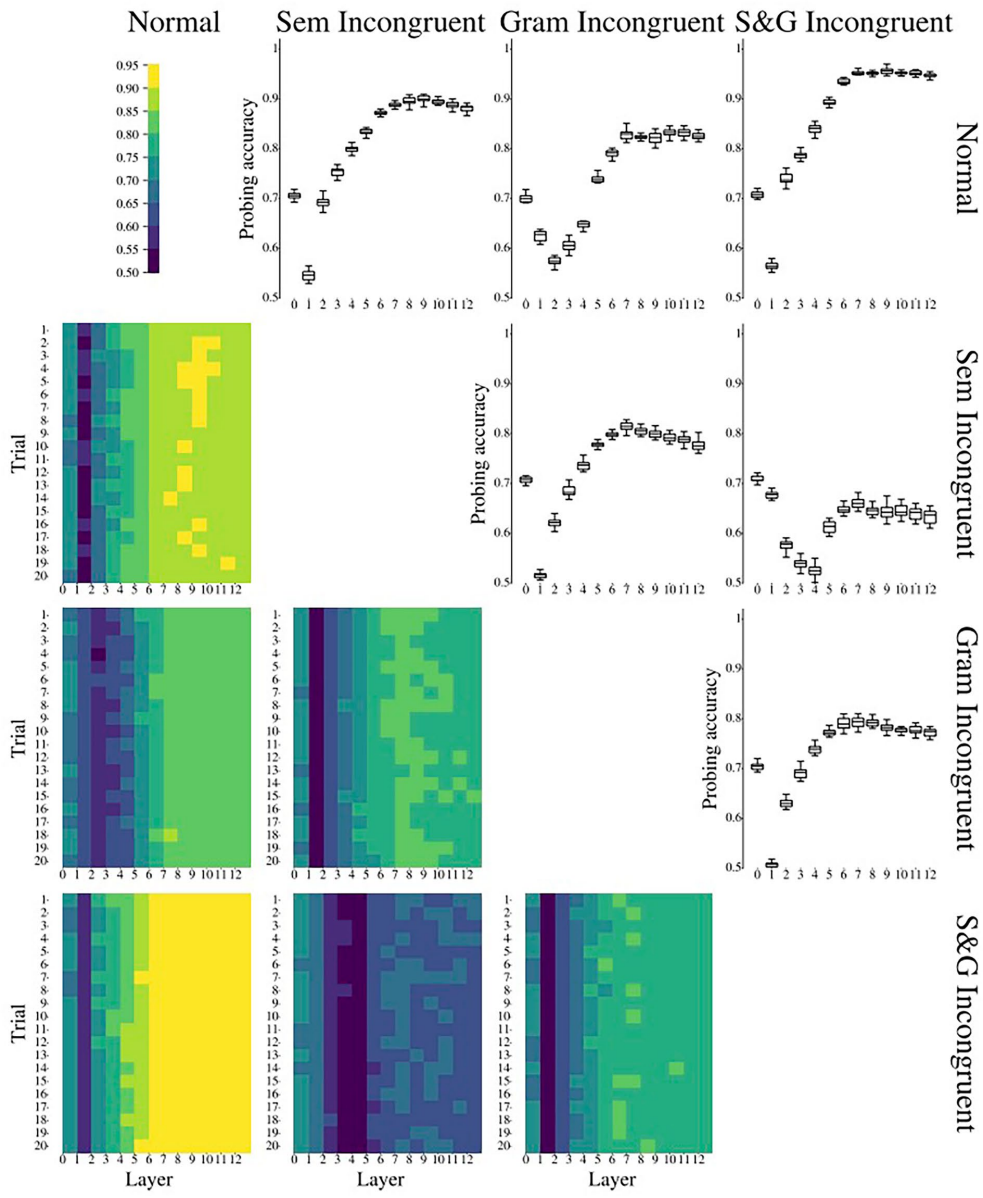
Results of layer-wise probing validation of the original dataset sampled from RuSentEval. Note. The lower matrix triangle represents the accuracy scores for the pairwise (in)congruence types classification obtained by applying logistic regression. The upper matrix triangle represents boxplots of the obtained accuracy scores; each boxplot shows the distribution of accuracy scores within 20 independent runs for each LLM layer and each pair of conditions. Normal – congruent condition. Sem Incongruent – semantically incongruent condition. Gram Incongruent – grammatically incongruent condition, S&G Incongruent – semantically-grammatically incongruent condition.

While probing experiments were performed with our proposed SIGNAL dataset, we also performed the same experimental pipeline with the sentences initially sampled from RuSentEval and their incongruent counterparts (see Fig. 10). Results obtained on this larger dataset supported our findings on SIGNAL dataset. Particularly, we found that semantic-grammatical incongruence was best distinguishable from congruent sentences, while semantically incongruent sentences were more surprising for the model than the grammatically incongruent ones.

### Summary of validation

Our work was aimed at developing a dataset containing linguistic stimulus data of sentences with four types of (in)congruence. To validate the stimulus material, we conducted a series of experiments to carefully select the stimuli, as well as to show that the difference between the incongruity (in)congruence can clearly revealed both in neuroimaging EEG data during human speech processing and for LLMs evident from neural activation data at each layer of the model.

In the initial online experiment we revealed that Russian native speakers could reliably distinguish the (in)congruence types of sentences at a behavioral level. Subsequent EEG validation confirmed statistically significant differences in ERPs across all pairs of conditions. Notably, the EEG data indicated that semantic and semantically-grammatical (in)congruence conditions elicited larger ERP differences compared to grammatical (in)congruence when contrasted with a congruent condition. An averaged ERP analysis identified a prominent negative deflection around 400 ms post-stimulus, consistent with the N400 effect commonly associated with semantic violations processing. Analogous to the EEG findings, LLMs probing experiments also revealed the most significant differences for semantic and semantic-grammatical (in)congruence, mirroring the validation results.

Therefore, the primary objective of this work was to present a stimuli dataset featuring four (in)congruence types and to confirm their clear distinguishability at both behavioral and neural levels in human language processing, as well as by LLMs. The validation experiments confirmed that semantic and semantic-grammatical (in)congruence yielded the most pronounced differences compared to congruent conditions, and to a greater extent than grammatical (in)congruence. Thus, the study successfully prepared and validated a stimulus material suitable for future theoretical research on human-model alignment.

## Supplementary information

Instructions for Toloka assessors

## Data Availability

The SIGNAL dataset presented in this study is publicly available at Hugging Face^35^: 10.57967/hf/6300. The stimuli data with the respective linguistic properties is provided in “.csv” format. The EEG data are available in the “.fif” and “.set” formats.

## Code availability

The custom pipelines of data generation and validation are available at https://github.com/AIRI-Institute/SIGNAL. Pipelines for stimuli generation are located in the “stimuli_generation” sub-directory. EEG data preprocessing and validation pipelines are located in the “eeg_processing” sub-directory. LLM probing pipelines are located in the “llm_processing” sub-directory.
