# Supplementary material for: SIGNAL: Dataset for Semantic and Inferred Grammar Neurological Analysis of Language
Source: Sci Data. 2025 Oct 24;12:1687. doi: 10.1038/s41597-025-05966-x (PMC12552490; doi:10.1038/s41597-025-05966-x)
Supplement: Supplementary file 1 — Instructions for Toloka assessors [file 41597_2025_5966_MOESM1_ESM.docx]

# Supplementary Information

# Instructions for Toloka assessors

Read the sentence. Assess if there is an error in this sentence. If there is one, specify its type.

**Types of errors**

- **The sentence is meaningless**: choose this type if you think that the sentences is meaningless, for example:

– *Avtobusy prohodjat massovuju fortunu.*

Buses are subject to massive fortune.

- **The sentence contains a grammatical error**: choose this option if one of the words in the sentence is in a wrong case or number, for examples:

– *Numeracija chashhe vsego proishodit natural’nymi chislom*.

Numbering most often occurs with natural number.

In this sentence a word is used in a wrong number, the correct way would be to use the word *chislami* ‘numbers’.

– *Posmotrite na nashih roditeli.*

Look at our parents.

In this example, the word is in the wrong case, the correct way of saying would be *na nashih roditelej* ‘at our parents’.

- **Hard to specify the error’s type**: if the sentence seems incorrect to you but the error is different from the errors described above, choose this option. Please choose this option only in the case if you are certain that this error cannot be classified with categories mentioned above.

Notice that a sentence can be fully acceptable, in this case you do not have to specify any type of an error. Moreover, sentences could contain both types of the errors:

– *Glavnyj geroj otpravljaetsja v trude.*

The main character is going to the hard work.

In this example, the word hard work does not fit into the sentence based on its meaning (it is not possible to go to *hard work*) and this word is in a wrong case (it should be accusative).
